# Novel method of real-time PCR-based screening for common fetal trisomies

**DOI:** 10.1186/s12920-021-01039-1

**Published:** 2021-07-30

**Authors:** So Yeon Kim, Seung Mi Lee, Sun Min Kim, Byoung Jae Kim, Ja Nam Koo, Ig Hwan Oh, Sohee Oh, Chan-Wook Park, Jong Kwan Jun, Ji Hyae Lim, Hyun Mee Ryu, Joong Shin Park

**Affiliations:** 1 Department of Obstetrics and Gynecology, Seoul National University College of Medicine, 101 Daehak-ro, Jongno-gu, Seoul, 03080 Korea; 2 Department of Obstetrics and Gynecology, University of Ulsan College of Medicine, Asan Medical Center, Seoul, Korea; 3 Department of Obstetrics and Gynecology, Seoul Metropolitan Government Seoul National University Boramae Medical Center, Seoul, Korea; 4 Seoul Women’s Hospital, Incheon, Korea; 5 Department of Biostatistics, Seoul Metropolitan Government Seoul National University Boramae Medical Center, Seoul, Korea; 6 Center for Prenatal Biomarker Research, CHA Advanced Research Institute, Gyeonggi-do, Korea; 7 Department of Obstetrics and Gynecology, CHA Bundang Medical Center, CHA University, 59 Yatap-ro, Bundang-gu, Seongnam-si, Gyeonggi-do, Korea

**Keywords:** Fetal trisomy, Prenatal diagnosis, Non-invasive prenatal test, Real-time polymerase chain reaction, Peptide nucleic acid

## Abstract

**Background:**

The non-invasive prenatal test (NIPT) is based on next generation sequencing (NGS) and is used for screening for fetal trisomy. However, it is time-consuming and technically difficult. Recently, peptide nucleic acid (PNA) probe-based real-time polymerase chain reaction (RT-PCR) was developed. This study aimed to examine the performance of the RT-PCR-based NIPT for screening of common fetal trisomies

**Methods:**

From stored maternal plasma, RT-PCR was performed using Patio™ NIPT Detection Kit. In melting curve analysis, the height of melting peaks of target chromosome and reference chromosome was calculated as a peak ratio. The adjusted peak ratio of 8 markers with correction factors in each target chromosome was summated and calculated to z-score. The cut-off value for each target chromosome was established for classification (low risk vs. high risk for trisomy) whose performance was obtained in the validation phase.

**Results:**

330 plasma samples from pregnant women with normal fetus and 22 trisomy cell-line samples were used to establish the optimal cut-off values for z-score of each target chromosome. In the validation phase, 1023 samples from pregnant women including 22 cases with fetal trisomy and 1001 cases of normal control were used. The RT-PCR-based NIPT showed 95.45% sensitivity [95% confidence interval (CI) 77.16–99.88%], 98.60% specificity (95% CI 97.66–99.23%), and 98.53% accuracy (95% CI 97.59–99.18%) for the identification of trisomy 21, 18, or 13. Of 1023 samples, fifteen cases were mismatched for classification [one case as a false negative (false negative rate: 4.5%) and 14 cases as false positives (false positive rate: 1.4%)].

**Conclusion:**

The RT-PCR-based NIPT showed high sensitivity and specificity for the detection of common fetal trisomies and it could be a feasible alternative to NGS-based NIPT.

**Supplementary Information:**

The online version contains supplementary material available at 10.1186/s12920-021-01039-1.

## Background

Screening strategies for common fetal trisomies of chromosomes 21, 18, and 13 have advanced considerably over the past few decades. The gold standard for fetal trisomy detection is karyotyping through invasive tests, such as amniocentesis and chorionic villus sampling. However, these methods carry a risk of complications for both the mother and the fetus.

After the discovery of cell-free fetal DNA (cffDNA) within maternal plasma by Lo et al. [1], noninvasive prenatal test (NIPT) is widely used as a screening test for aneuploidy due to its high sensitivity and specificity, despite the shortcomings such as placental mosacism and test failure [2]. Various non-invasive methods of prenatal screening for common fetal trisomies have been suggested, usually based on the next generation sequencing (NGS) [3, 4]. However, such methods are often technically challenging, time-consuming, and require specialized equipment. Furthermore, the optimal method for incorporating cffDNA screening into existing programs continues to be debated [5, 6].

Recently, molecular techniques based on the peptide nucleic acid (PNA) probe-based real-time polymerase chain reaction (RT-PCR) have been proposed for identifying chromosomal abnormalities and detecting genetic variation. PNA probes introduce a large difference in melting temperatures between perfectly matched and mismatched sequences, allowing this method to be widely applied in molecular biology [7–9]. For example, PNA probes have been used in the detection of clarithromycin resistance in *Helicobacter pylori* and microsatellite instability in colorectal carcinoma [7, 10–12]. Additionally, this technology only needs RT-PCR equipment, making it relatively easy to operate, quick, and cost-effective [13].

Therefore, in this study, we examined the performance of a non-invasive prenatal test (NIPT) that utilized PNA probe-based RT-PCR as an alternative to the conventional NGS-based NIPT to screen for common fetal trisomies.

## Methods

### Study design

In the development phase, the cut-off value for the z-score in the RT-PCR-based NIPT was established for classification (low risk vs. high risk for trisomy). For this, 330 plasma samples from pregnant women with normal fetuses and 22 trisomy cell line samples purchased from the Coriell Cell Repositories (GM01137, GM01413, GM02504, GM02571, GM03606, GM04616, GM04617, GM04965, AG05121, AG11552; GM00734, GM02732, GM03538, GM03623, GM20912, AG07167, AG0801; GM00503, GM00526, GM02948, GM03330, AG12070) were used to establish the optimal cut-off values for the z-score of each target chromosome. In the validation phase, the performance of the screening method using PNA probe-based RT-PCR was validated with 1,023 plasma samples from pregnant women including 22 cases with common aneuploidies and 1,001 normal control cases.

**Fig. 1:**
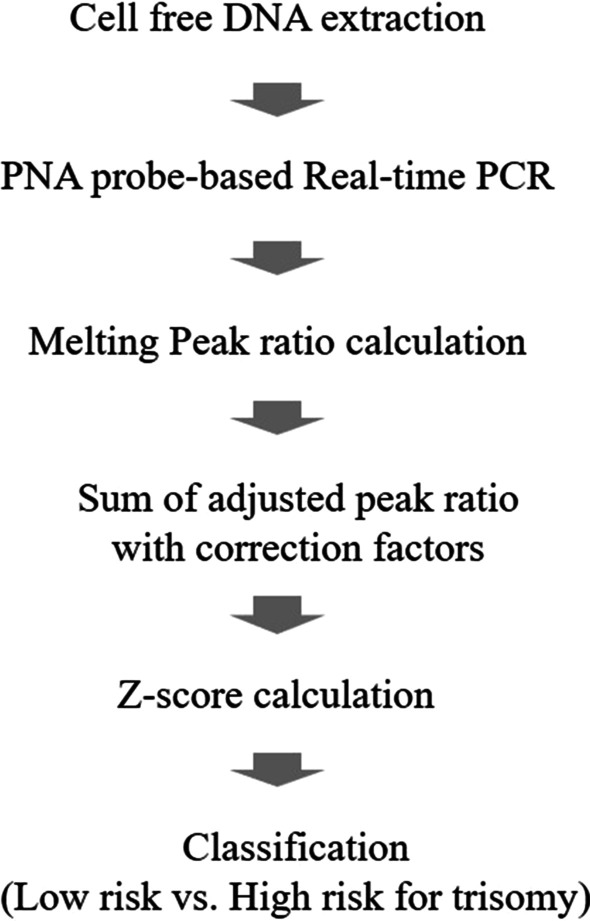
Brief flowchart of the examination process. Peptide nucleic acid (PNA)

The stored maternal plasma samples were obtained from five different institutions (Seoul National University Hospital, Seoul Metropolitan Government Seoul National University Boramae Medical Center, Seoul Women’s Hospital, CHA Bundang Medical center, and Pusan National University Hospital; all in the Republic of Korea). These institutions prospectively collected plasma samples of pregnant women who gave written consent for their clinical information and biologic samples to be used for research purposes. Maternal plasma samples were used for PNA probe-based RT-PCR, and the classification afforded by the RT-PCR-based NIPT was compared to the previously established results. Brief process of the RT-PCR-based NIPT is shown in Fig. 1. This study was approved by the Institutional Review Board and was conducted according to the ethical principles expressed in the Declaration of Helsinki.

### Sample preparation

All samples were taken by venipuncture and transferred into an ethylenediaminetetraacetic acid (EDTA)-containing tube, and then stored at − 70 ℃ after centrifugation. The cell-free DNA was extracted from the stored maternal plasma using a QIAamp Circulating Nucleic Acid Kit (Qiagen, Hilden, Germany) according to the manufacturer’s instructions.

### Screening kit for the detection of fetal aneuploidy

**Fig. 2:**
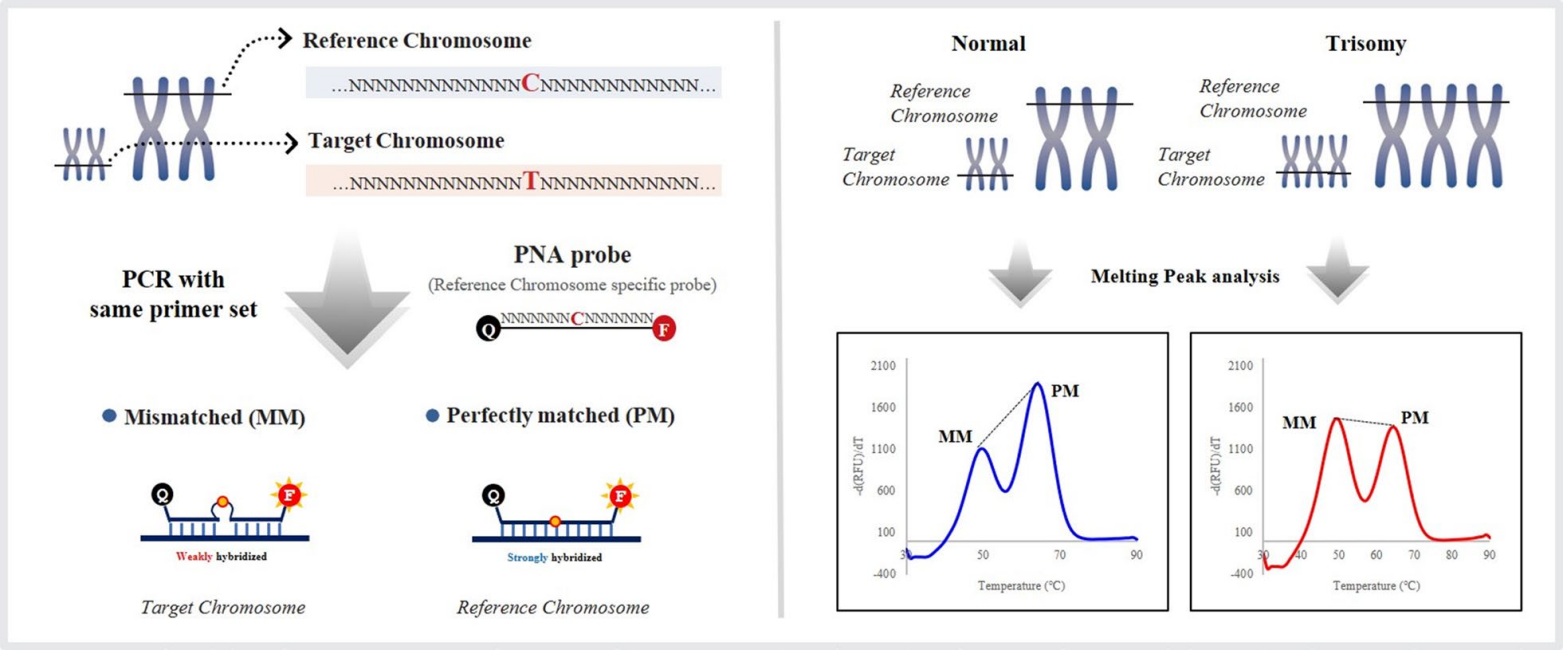
The principles of PNA probe-based RT-PCR combined with melting curve analysis. Peptide nucleic acid (PNA)

A Patio™ NIPT Detection Kit (SeaSun Biomaterials, Daejeon, Korea) consisted of 2 × qPCR premix, primer & PNA probe mixture (NIPT set 1–12) and standard control DNA 1–3 (Reference control DNA). PNA probes were designed to hybridize to paralogous sequences of the target chromosome and reference chromosome, which were nearly identical, differing in one or two nucleotides. This resulted in a difference of melting points between target chromosome and reference chromosome (Fig. 2). Eight paralogous sequences (DNA markers) and corresponding PNA probes were used for each target chromosome, and the PNA probes were fluorescently labeled with fluorescein amidite or hexachloro-fluorescein.

Due to a minute difference in DNA amplicons (PCR products) for fetal trisomy analysis, robust, highly accurate, and precise methods were required. The kit included an additional probe that eliminated part of the total amplicon for maximizing analytical sensitivity. There was an apparent competitive relationship between the detection probe and additional probe concerning the paralogous sequence in optimized conditions (ex. PCR cycles, time, and temperature of denaturation/annealing/extension, and probe concentration). Assuming fetal fraction of 10%, the difference in the ratio between normal and trisomy cases was 1:1.05; however, additional probes could increase this difference up to tenfold (1:1.5). The primer and probe sequences could not be revealed due to the manufacturer’s (SeaSun Biomaterials) patent right.

### Real-time PCR and melting curve analysis

RT-PCR (Bio-Rad, Hercules, CA, USA) was performed in a final volume of 20 μL that contained 10 μL of 2 × qPCR PreMix, 7 μL of primer and PNA probe mixture (Patio ™ NIPT Detection Kit, NIPT set 1–12), and 3 μL of the cell free DNA template. The reaction conditions for the amplification were 50 °C for 5 min, 95 °C for 10 min, and 60 cycles of [95 °C for 30 s, 58 °C for 45 s, and 72 °C for 45 s] followed by melting curve analysis. The latter was performed using a denaturation step of 95 °C for 5 min; 1 min hybridization steps of 75 °C, 55 °C, and 45 °C; and a stepwise temperature increase from 30 to 90 °C at 1 °C/step, with a 5 s interval between each step. The data were analyzed using Bio-Rad CFX Manager v3.0 software (Bio-Rad, Hercules, CA, USA).

### Interpretation of results

In the melting curve analysis, the heights of melting peaks of the target and reference chromosomes were used for peak ratio calculation, which reflected the relative ratio of the amounts of each chromosome. The adjusted peak ratio of eight markers with correction factors in each target chromosome was summated and expressed as z-score.

### Confirmation of fetal aneuploidies

Fetal trisomies were confirmed by an invasive prenatal test, such as amniocentesis or chorionic villus sampling. The normal karyotype was either determined by the invasive test or considered as normal if clinically normal phenotype was observed after birth.

### Statistical analysis

To assess the predictive ability of the Patio™ NIPT Detection Kit, the values of sensitivity, specificity, positive predictive value, negative predictive value, and diagnostic accuracy with 95% CI were calculated. Comparison of the continuous variables was performed by using the Mann–Whitney U test. All statistical analyses were performed by using MedCalc Statistical Software version 19.0.5 (MedCalc Software bvba, Ostend, Belgium; https://www.medcalc.org; 2019) and R version 3.6.1 (http://www.r-project.org). Differences were considered statistically significant when *P* < 0.05.

## Results

### Determination of the optimal cut-off values for the z-score of each target chromosome

In the development phase, ten trisomy 21, seven trisomy 18, and five trisomy 13 cell lines and 330 maternal plasma samples as normal control group were used for determining the optimal cut-off values. PNA probe-based RT-PCR was performed, and adjusted peak ratio of eight markers with correction factors in each target chromosome was summated and expressed as the z-score (Additional file 1: Table 1). The cut-off value of the z-score was determined to include all of the trisomy samples but exclude those with normal karyotype. The cut-off value was 3 for Down syndrome, 2 for Edward syndrome, and 1 for Patau syndrome.

### Validation of RT-PCR-based NIPT performance

After determining the cut-off value, 1,023 samples were analyzed as a validation set, which included 22 cases with fetal trisomy and 1,001 cases of normal controls. Demographic details of the study group are provided in Table 1. The median maternal age was 33 years (30–36 years; interquartile range), and median gestational age at sampling was 12.6 weeks (12.1–13.3 weeks; interquartile range). The body mass index (BMI) was calculated from weight and height of the subjects. The median BMI was 21.5 (19.9–24.3; interquartile range). The 22 cases of fetal aneuploidy (fourteen of trisomy 21, five of trisomy 18, and three of trisomy 13) were confirmed by invasive tests.

**Table. 1:** Demographic characteristics of the study cohort

Characteristics	Values
Age, years	33 (30–36) (n = 1023)
Height, cm	161.3 (158.0–165.0) (n = 1020)
Weight at sampling, kg	56.5 (51.8–63.9) (n = 1000)
BMI at sampling	21.5 (19.9–24.3) (n = 998)
GA at sampling, weeks	12.6 (12.1–13.3) (n = 1023)

Data are presented as median (interquartile range); BMI, body mass index; GA, gestational age

**Fig. 3:**
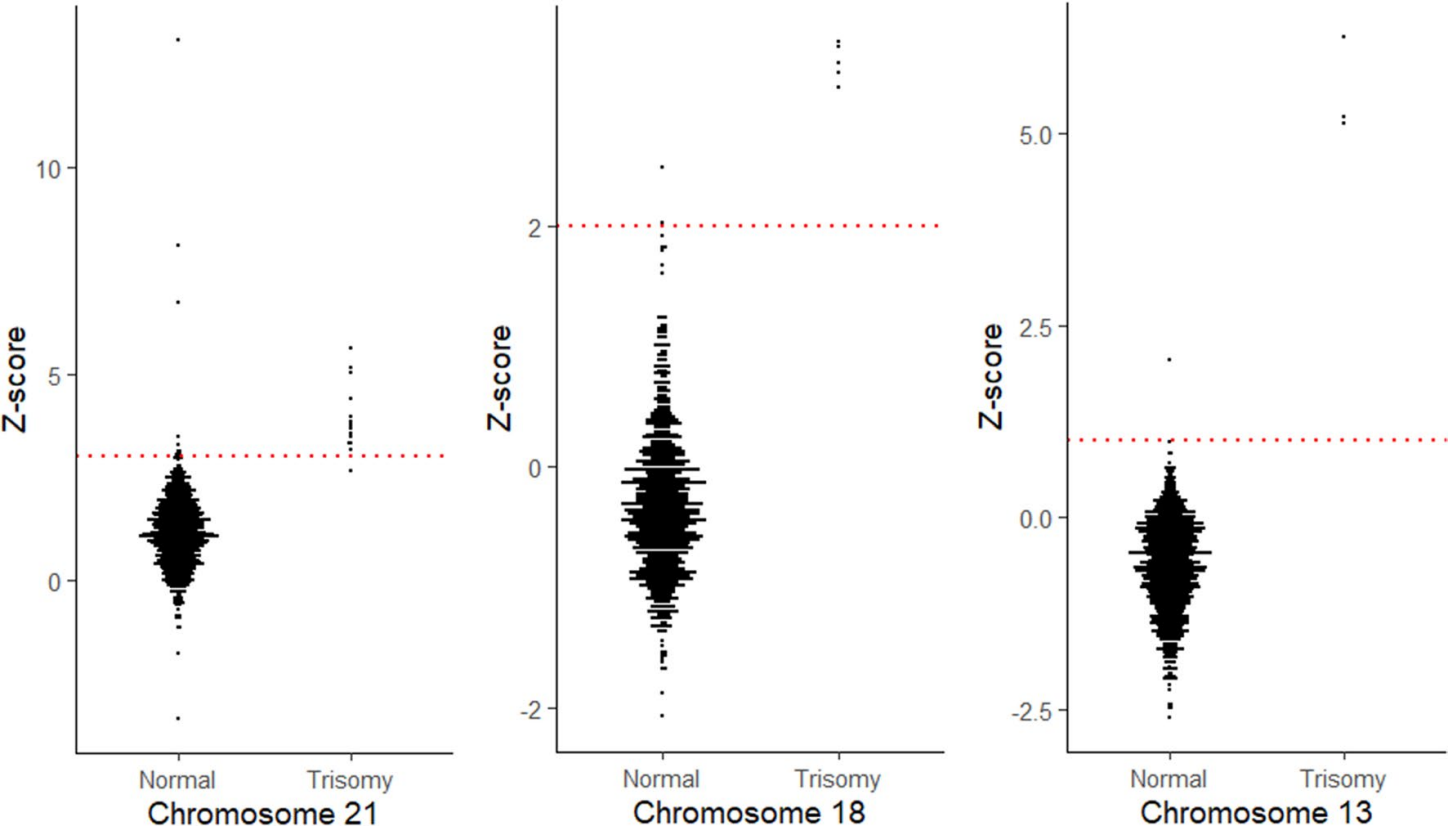
Z-score distributions for each fetal aneuploidy (trisomy 21, 18, 13) with the cut-off value

Figure 3 shows z-score distributions of each fetal chromosome (chromosomes 21, 18, and 13). There were no cases with test failure (i.e., invalid test results). The z-score of chromosome 21 showed significant differences in the distribution between normal and aneuploidy fetal karyotype cases (median z-score 1.12 vs. 3.72, *P* < 0.001). Among the 14 cases with fetal trisomy 21, the RT-PCR-based NIPT classified 13 cases as high risks for trisomy 21 (92.86% [13/14]), when the pre-established cut-off value (> 3) was used. The RT-PCR-based NIPT also sensitively detected fetal trisomy 18 and trisomy 13 (median z-score in chromosome 18, − 0.38 vs. 3.36, *P* < 0.001; median z-score in chromosome 13, − 0.62 vs. 5.21, *P* < 0.005). The use of a cut-off value for each chromosome enabled all cases of trisomies 18 and 13 to be classified as high risk for respective trisomies.

Table 2 shows the predictive performance of the RT-PCR-based NIPT. The values of the sensitivity to detect fetal trisomies 21, 18, and 13 were 92.86% (95% confidence interval [CI] 66.13–99.82%), 100% (95% CI 47.82–100.00%), and 100% (95% CI 29.24–100.00%), respectively. The values of the specificity to detect fetal trisomies 21, 18, and 13 were 98.91% (95% CI 98.06–99.46%), 99.80% (95% CI 99.29–99.98%), and 99.90% (95% CI 99.45–100.00) respectively. Among 1,001 euploid samples, 14 were falsely classified as high risk for any trisomy. Details of trisomy and misclassified cases are provided in Electronic Additional file 1: Tables 2 and 3, respectively. The overall classification showed 95.45% sensitivity (95% CI 77.16–99.88%), 98.60% specificity (95% CI 97.66–99.23%), and 98.53% accuracy (95% CI 97.59–99.18%).

**Table. 2:** Sensitivity and specificity of the detection of common fetal trisomies

	Down syndrome	Edward syndrome	Patau syndrome	Overall
Sensitivity, %	92.86 (66.13–99.82)	100 (47.82–100.00)	100 (29.24–100.00)	95.45 (77.16–99.88)
Specificity, %	98.91 (98.06–99.46)	99.80 (99.29–99.98)	99.90 (99.45–100.00)	98.60 (97.66–99.23)
LR +	85.18 (46.49–156.04)	509.00 (127.47–2032.43)	1020.00 (143.82–7234.10)	68.25 (40.25–115.73)
LR–	0.07 (0.01–0.48)	0.00 (0.00–NaN)	0.00 (0.00–NaN)	0.05 (0.01–0.31)
PPV, %	54.17 (32.82–74.45)	71.43 (29.04–96.33)	75.00 (19.41–99.37)	60.00 (42.11–76.13)
NPV, %	99.90 (99.44–100.00)	100.00 (99.64–100.00)	100.00 (99.64–100.00)	99.90 (99.44–100.00)
Accuracy, %	98.83 (97.96–99.39)	99.80 (99.30–99.98)	99.90 (99.46–100.00)	98.53 (97.59–99.18)

Data in parentheses are 95% confidence intervals; LR + , positive likelihood ratio; LR-, negative likelihood ratio; PPV, positive predictive value; NPV, negative predictive value; NaN, not-a-number

### Superior performance of the RT-PCR-based NIPT compared to that of other methods

In our study cohort, 924 participants underwent conventional screening test (one participant had an unknown result), 51 participants underwent NGS-based NIPT (currently commercialized), and 47 participants initially underwent invasive diagnostic tests. One participant did not receive any other screening tests. Table 3 shows comparison of the results of the conventional tests with those of RT-PCR-based NIPT. Specificity of the RT-PCR-based NIPT was significantly higher than of the non-NIPT screening test but similar to that of the NGS-based NIPT.

**Table. 3:** Comparison of the results of the conventional test with those of the RT-PCR-based NIPT

	Non-NIPT screening test^a^ (n = 924)		RT-PCR-based NIPT (n = 924)		P
Sensitivity	5/5	100%	5/5	100%	(–)
Specificity	834/918	91%	905/918	99%	< 0.0001
Screening positive rate	89/924	10%	18/924	1.9%	

	NGS based NIPT (n = 51)		RT-PCR-based NIPT (n = 51)		P
Sensitivity	3/3	100%	3/3	100%	(–)
Specificity	48/48	100%	47/48	98%	NS
Screening positive rate	3/51	6%	4/51	8%	

	Diagnostic test or no screening test^b^ (n = 48)	RT-PCR-based NIPT (n = 48)		P
Sensitivity	N/A	12/13	92%	(–)
Specificity	N/A	35/35	100%	(-)
Screening positive rate	N/A	12/48	25%	

NIPT, non-invasive prenatal test; RT-PCR, real-time polymerase chain reaction; NGS, next generation sequencing; N/A, not applicable; NS, non-specific

^a^Including 2 nuchal translucency, 14 Quad tests, 905 integrated tests, and 3 sequential tests (one had a sequential test, but the result was unknown)

^b^47 performed an invasive diagnostic test as the primary test while 1 patient did not undergo screening test

## Discussion

The current study demonstrated excellent performance of the RT-PCR-based NIPT in screening for common fetal trisomies. The overall performance of the NIPT using PNA probe-based RT-PCR in screening for fetal trisomies 21, 13, and 18 showed 95.45% sensitivity, 98.60% specificity, and no test failures.

PNAs, which are DNA analogs, are artificially synthesized with uncharged backbone and, therefore, have more favorable hybridization properties as well as chemical, thermal, and biological stability parameters [10, 14]. The PNA probe composed of dual-labeled (quencher and fluorophore) to improve the resolution of detection, causes a large difference in melting temperature between specific hybridization and partial hybridization. PNAs are becoming increasingly used in different molecular biology applications [7–9], e.g., in the detection of clarithromycin resistance in *Helicobacter pylori* or demonstration of microsatellite instability in colorectal carcinoma [7, 10–12].

In obstetrics, we have reported that PNA probes could be used for rapid determination of aneuploidy in amniotic fluid samples, as an alternative to fluorescence in situ hybridization, quantitative fluorescence PCR, and multiplex ligation-dependent probe amplification [7]. In the current study, we also demonstrated that PNA probe-based RT-PCR NIPT performed superbly in the screening for common fetal aneuploidy conditions.

The NIPT based on the analysis of cell-free maternal plasma DNA is an innovative approach to screening for common fetal aneuploidies [3, 4]. Numerous studies have shown that NIPT detects common fetal trisomies with high sensitivity and specificity. However, the NIPT analyses the placental DNA, not real fetal DNA, and might detect vanishing twin, maternal malignancy and maternal mosaicism, etc. [15]. In addition, until now, most NIPT studies have been based on NGS and therefore, reported frequent test failures up to 5% of the -when cell-free DNA concentration or fetal fraction was low [16–19]. RT-PCR-based NIPT can report the result with smaller amounts of maternal blood samples than conventional methods. For example, most existing NIPT methods require a minimum of 10 mL of maternal blood sample to perform the test, whereas just 6 mL is sufficient with the method used in our study. Both RT-PCR- and NGS-based NIPTs include PCR amplification of cell free DNA; however, the RT-PCR-based method has a single amplification step with fewer amplified region than the NGS-based method. Therefore, RT-PCR-based NIPT enables a relatively more stable amplification process with a smaller blood sample volume.

After the discovery of cffDNA, non-invasive prenatal testing using NGS methods has rapidly developed. However, the NGS approach requires expensive equipment, reagents, and software, and has limited throughput [5]. In addition, the result turnaround time is long.

Several other techniques, e.g., identification of the methylated regions, or plasma microRNAs, have been suggested for the NIPT [20–22]. The methylated region approaches needed pretreatment with sodium bisulfite or methylation-sensitive restriction enzymes. The former process has a low reproducibility, and the latter process requires long processing. Moreover, the plasma microRNAs approaches might be difficult to perform at early pregnant due to very low levels of microRNAs. Because of such limitations, these methods are not commonly used.

Compared with the features of the NGS-based approach, RT-PCR-based NIPT has rapid, easy-handling, and low-cost procedures for screening of common fetal trisomies. The RT-PCR-based NIPT provides the results within 6 h, i.e., much easier, and faster than other molecular methods, such as NGS, which require long turnaround time and labor-intensive experiments.

In this study, 15 out of 1,023 samples were misclassified (Additional file 1: Table 3). Fourteen cases were classified as false positives. No significant differences in the characteristics between the correctly classified and falsely classified groups in normal karyotype cases were observed (data not shown). However, one case was misclassified as false negative for trisomy 21, gestational age at sampling of false negative case was 12^+4^ weeks and BMI at sampling was 34.9. There may be several reasons for misclassification by the NIPT based on cell-free DNA, including confined placental or real fetal mosaicism, vanishing twins, maternal somatic mosaicism, maternal copy number variants, or undetected maternal cancer [23]. In addition, after visually inspecting the dotplots of 330 maternal plasma samples of normal fetuses and 22 trisomy cell line samples, the cut-off value for classification was determined as the value best separating the two groups. However, validating this cut-off value to our larger dataset yielded a few overlapped samples resulting in false positives and negatives. To be used in clinical practice, more larger studies may be needed to estimate actual performance in real-world.

To the best of our knowledge, this is the first study that utilized PNA probe-based RT-PCR for a NIPT. However, our study has limitations in that it was a retrospective examination of stored maternal samples and electronic medical records from five different institutions. Furthermore, only pregnant women of the Asian race (mostly Korean) were included. Due to the retrospective study design, the performance of RT-PCR-based NIPT presented in the current study may not represent the real-world data both in low risk and high risk pregnancies. Therefore, additional prospective studies with larger study cohorts, including participants of various races, are needed for the validation of this NIPT in real clinical practice. In addition, a comparative analysis of the sensitivity and cost-effectiveness of RT-PCR-based and NGS-based NIPTs is needed. While the NIPT only provides information on common trisomies such as trisomy 18, 13, and 21, the first trimester screening test (FTS) conducted in the same period may provide additional information on various genetic abnormalities. Therefore, further study is needed to compare the performance between NIPT and FTS for various genetic diseases. The novel method could technically distinguish the microdeletion/microduplication syndrome from normal control to create PNA probes for targeting subchromosomes; however, the incidence of subchromosomal abnormality is very low, and the actual practicality and applicability of its efficacy would be difficult to prove. Therefore, it is necessary to extend the range of NIPTs from trisomies of chromosome 21, 18, and 13 to other aneuploidy conditions and subchromosomal abnormalities.

## Conclusion

In conclusion, the PNA probe-based RT-PCR showed high sensitivity and specificity for the detection of fetal common aneuploidies. This method could be a feasible alternative to the NGS in the cffDNA screening test.

## Supplementary Information

**Additional file 1**. **Supplementary Table 1**. Details of target chromosome DNA markers. FAM, Fluorescein amidite; HEX, Hexachloro-fluorescein; DS, Down syndrome; ES, Edward syndrome; PS, Patau syndrome; PM, perfect match temperature; MM, mismatch temperature; CF, correction factor; SD, standard deviation.** Supplementary Table 2**. Details of fetal trisomy cases. BMI, body mass index; GA, gestational age; CVS, chorionic villi sampling.** Supplementary Table 3**. Details of false positive and false negative cases. * Absence of common fetal trisomies was confirmed using stored cord blood which was sampled at a delivery; † No result was obtained with QF-PCR (test failure d/t low DNA amount); BMI, body mass index; GA, gestational age; QF-PCR, quantitative fluorescent polymerase chain reaction.

## Data Availability

The datasets analysed during the current study are available in the figshare repository, https://doi.org/10.6084/m9.figshare.14582805.

## Abbreviations

PCR: Polymerase chain reaction
NIPT: Non-invasive prenatal test
NGS: Next generation sequencing
PNA: Peptide nucleic acid
RT-PCR: Real-time polymerase chain reaction
cffDNA: Cell-free fetal DNA
FTS: First trimester screening test
