# Supplementary material for: Novel method of real-time PCR-based screening for common fetal trisomies
Source: BMC Med Genomics. 2021 Jul 30;14:195. doi: 10.1186/s12920-021-01039-1 (PMC8323267; doi:10.1186/s12920-021-01039-1)
Supplement: Supplementary file 1 — Additional file 1. Supplementary Table 1. Details of target chromosome DNA markers. FAM, Fluorescein amidite; HEX, Hexachloro-fluorescein; DS, Down syndrome; ES, Edward syndrome; PS, Patau syndrome; PM, perfect match temperature; MM, mismatch temperature; CF, correction factor; SD, standard deviation. Supplementary Table 2. Details of fetal trisomy cases. BMI, body mass index; GA, gestational age; CVS, chorionic villi sampling. Supplementary Table 3. Details of false positive and false negative cases. * Absence of common fetal trisomies was confirmed using stored cord blood which was sampled at a delivery; † No result was obtained with QF-PCR (test failure d/t low DNA amount); BMI, body mass index; GA, gestational age; QF-PCR, quantitative fluorescent polymerase chain reaction. [file 12920_2021_1039_MOESM1_ESM.docx]

**Novel method of real-time PCR-based screening**

**for common fetal trisomies**

So Yeon Kim^1,2^, Seung Mi Lee^1^, Sun Min Kim^1,3^, Byoung Jae Kim^1,3^, Ja Nam Koo^4^, Ig Hwan Oh^4^, Sohee Oh^5^, Chan-Wook Park^1^, Jong Kwan Jun^1^, Ji Hyae Lim^6^, Hyun Mee Ryu^6,7^, Joong Shin Park^1^

^1^Department of Obstetrics and Gynecology, Seoul National University College of Medicine, Seoul, Korea

^2^Department of Obstetrics and Gynecology, University of Ulsan College of Medicine, Asan Medical Center, Seoul, Korea

^3^Department of Obstetrics and Gynecology, Seoul Metropolitan Government Seoul National University Boramae Medical Center, Seoul, Korea

^4^Seoul Women's Hospital, Incheon, Korea

^5^Department of Biostatistics, Seoul Metropolitan Government Seoul National University Boramae Medical Center, Seoul, Korea

^6^Center for Prenatal Biomarker Research, CHA Advanced Research Institute, Gyeonggi-do, Korea

^7^Department of Obstetrics and Gynecology, CHA Bundang Medical center, CHA University, Gyeonggi-do, Korea

This study was presented at the 30th Virtual World Congress on Ultrasound in Obstetrics and Gynecology (ISUOG), October 16-18, 2020.

SY Kim and SM Lee contributed equally as the first authors of this study.

JS Park and HM Ryu contributed equally as co-corresponding authors.

**Correspondence to:**

Joong Shin Park, MD, PhD

Department of Obstetrics and Gynecology

Seoul National University College of Medicine

101 Daehak-ro, Jongno-gu, Seoul 03080, Korea.

E-mail: [jsparkmd@snu.ac.kr](mailto:jsparkmd@snu.ac.kr)

Tel: +82-2-2072-3199

Fax: +82-2-762-3599

Hyun Mee Ryu, MD, PhD

Department of Obstetrics and Gynecology

CHA Bundang Medical Center

59 Yatap-ro, Bundang-gu, Seongnam-si, Gyeonggi-do, Korea

E-mail: hmryu@cha.ac.kr

Tel: +82-31-780-5290

Fax: +82-31-780-5960

**Supplementary Table 1: Details of target chromosome DNA markers**

| Tube | Fluorescent | Marker | MMT | PMT | Ratio | CF | Average | SD | Cut-off |
| --- | --- | --- | --- | --- | --- | --- | --- | --- | --- |
| NIPT-1 | FAM | DS-1 | 50± 3℃ | 65± 3℃ | MM/PM | 1.89 | 7.98 | 0.609 | > 3 |
|  | HEX | DS-2 | 58± 3℃ | 71± 3℃ | MM/PM | 1.58 |  |  |  |
| NIPT-2 | FAM | DS-3 | 50± 3℃ | 68± 3℃ | MM/PM | 1.77 |  |  |  |
|  | HEX | DS-4 | 65± 3℃ | 74± 3℃ | MM/PM | 1.84 |  |  |  |
| NIPT-3 | FAM | DS-5 | 57± 3℃ | 71± 3℃ | MM/PM | 1.38 |  |  |  |
|  | HEX | DS-6 | 59± 3℃ | 69± 3℃ | MM/PM | 1.36 |  |  |  |
| NIPT-4 | FAM | DS-7 | 54± 3℃ | 70± 3℃ | MM/PM | 1.41 |  |  |  |
|  | HEX | DS-8 | 50± 3℃ | 71± 3℃ | MM/PM | 1.9 |  |  |  |
| NIPT-5 | FAM | ES-1 | 56± 3℃ | 68± 3℃ | MM/PM | 1.85 | 7.96 | 0.728 | > 2 |
|  | HEX | ES-2 | 55± 3℃ | 67± 3℃ | PM/MM | 1.26 |  |  |  |
| NIPT-6 | FAM | ES-3 | 52± 3℃ | 76± 3℃ | MM/PM | 1.21 |  |  |  |
|  | HEX | ES-4 | 57± 3℃ | 68± 3℃ | PM/MM | 1.32 |  |  |  |
| NIPT-7 | FAM | ES-5 | 52± 3℃ | 66± 3℃ | MM/PM | 1.37 |  |  |  |
|  | HEX | ES-6 | 56± 3℃ | 69± 3℃ | PM/MM | 1.26 |  |  |  |
| NIPT-8 | FAM | ES-7 | 59± 3℃ | 68± 3℃ | MM/PM | 1.35 |  |  |  |
|  | HEX | ES-8 | 51± 3℃ | 60± 3℃ | PM/MM | 1.18 |  |  |  |
| NIPT-9 | FAM | PS-1 | 64± 3℃ | 75± 3℃ | MM/PM | 1.32 | 8.47 | 0.571 | > 1 |
|  | HEX | PS-2 | 50± 3℃ | 67± 3℃ | PM/MM | 1.35 |  |  |  |
| NIPT-10 | FAM | PS-3 | 61± 3℃ | 75± 3℃ | MM/PM | 1.66 |  |  |  |
|  | HEX | PS-4 | 48± 3℃ | 63± 3℃ | PM/MM | 1.37 |  |  |  |
| NIPT-11 | FAM | PS-5 | 55± 3℃ | 70± 3℃ | MM/PM | 1.58 |  |  |  |
|  | HEX | PS-6 | 48± 3℃ | 63± 3℃ | PM/MM | 1.25 |  |  |  |
| NIPT-12 | FAM | PS-7 | 57± 3℃ | 69± 3℃ | MM/PM | 1.46 |  |  |  |
|  | HEX | PS-8 | 55± 3℃ | 69± 3℃ | MM/PM | 1.44 |  |  |  |

FAM, Fluorescein amidite; HEX, Hexachloro-fluorescein; DS, Down syndrome; ES, Edward syndrome; PS, Patau syndrome; PM, perfect match temperature; MM, mismatch temperature; CF, correction factor; SD, standard deviation

**Supplementary Table 2. Details of fetal trisomy cases**

| Sample No. | Karyotype | Maternal age, years | Height  , cm | Weight  , kg | BMI | GA at  blood sampling | Confirmation test |
| --- | --- | --- | --- | --- | --- | --- | --- |
| 1 | 47, XN, +21 | 35 | 162.9 | 54.7 | 20.6 | 16.4 | amniocentesis |
| 2 | 47, XN, +21 | 35 | 153 | 57 | 24.3 | 12.7 | amniocentesis |
| 3 | 47, XN, +21 | 38 | 158.1 | 55.2 | 22.1 | 13.4 | CVS |
| 4 | 47, XN, +21 | 39 | 158 | 53.1 | 21.3 | 12 | CVS |
| 5 | 47, XN, +21 | 40 | 170 | 101 | 34.9 | 12.6 | amniocentesis |
| 6 | 47, XN, +21 | 45 | 150 | 60 | 26.7 | 12.7 | CVS |
| 7 | 47, XN, +21 | 35 | 157 | 50 | 20.3 | 13.6 | CVS |
| 8 | 47, XN, +21 | 40 | 161 | 63.9 | 24.7 | 16.7 | amniocentesis |
| 9 | 47, XN, +21 | 33 | 169 | 60.9 | 21.3 | 20.9 | amniocentesis |
| 10 | 47, XN, +21 | 39 | 164 | 51 | 19.0 | 16.9 | amniocentesis |
| 11 | 47, XN, +21 | 34 | 168 | 58 | 20.5 | 20.4 | amniocentesis |
| 12 | 47, XN, +21 | 36 | 161 | 58.5 | 22.6 | 15.7 | CVS |
| 13 | 47, XN, +21 | 41 | 155 | 49.1 | 20.4 | 16.6 | amniocentesis |
| 14 | 47, XN, +21 | 37 | 153 | 45 | 19.2 | 16.1 | amniocentesis |
| 15 | 47, XN, +18 | 40 | 160 | 63.3 | 24.7 | 11.9 | CVS |
| 16 | 47, XN, +18 | 40 | 168 | 62 | 22.0 | 16 | amniocentesis |
| 17 | 47, XN, +18 | 37 | 152 | 43.5 | 18.8 | 18.9 | amniocentesis |
| 18 | 47, XN, +18 | 33 | 158 | 44.2 | 17.7 | 22.3 | amniocentesis |
| 19 | 47, XN, +18 | 39 | 162 | 54 | 20.6 | 15.3 | amniocentesis |
| 20 | 47, XN, +13 | 31 | 163.9 | 54.9 | 20.4 | 16.4 | CVS |
| 21 | 47, XN, +13 | 40 | 169 | 54 | 18.9 | 15.9 | amniocentesis |
| 22 | 47, XN, +13 | 36 | 165 | 76 | 27.9 | 13.7 | amniocentesis |

BMI, body mass index; GA, gestational age; CVS, chorionic villi sampling

**Supplementary Table 3. Details of false positive and false negative cases**

| Sample No. | NIPT  Result  or karyotype | | Maternal age, years | Height  , cm | | Weight  , kg | BMI | GA at  blood sampling | Confirmation test |
| --- | --- | --- | --- | --- | --- | --- | --- | --- | --- |
| False Positive Cases | |  | | |  |  |  |  |  |
| 1 | Down high risk | | 26 | 161.6 | | 64.0 | 24.5 | 31.1 | Amniocentesis |
| 2 | Down high risk | | 38 | 168.4 | | 69.0 | 24.3 | 30.4 | Physical exam |
| 3 | Down high risk | | 28 | 153.0 | | 36.0 | 15.4 | 13.6 | QF-PCR* |
| 4 | Down high risk | | 28 | 165.0 | | 56.0 | 20.6 | 12.6 | QF-PCR* |
| 5 | Down high risk | | 27 | 160.0 | | 54.1 | 21.1 | 12.1 | QF-PCR* |
| 6 | Down high risk | | 32 | 156.0 | | 61.0 | 25.1 | 12.6 | QF-PCR* |
| 7 | Down high risk | | 36 | 155.0 | | 46.3 | 19.3 | 12.6 | QF-PCR* |
| 8 | Down high risk | | 36 | 162.0 | | 55.6 | 21.2 | 12.3 | QF-PCR* |
| 9 | Down high risk | | 38 | 159.0 | | 70.6 | 27.9 | 12.1 | Physical exam† |
| 10 | Down high risk | | 35 | 153.0 | | 49.5 | 21.1 | 12.9 | QF-PCR* |
| 11 | Down high risk | | 36 | 163.0 | | 54.0 | 20.3 | 12.0 | Physical exam |
| 12 | Edward high risk | | 35 | 158.6 | | 42.5 | 16.9 | 11.3 | Physical exam |
| 13 | Edward high risk | | 26 | 154.0 | | 49.0 | 20.7 | 12.3 | QF-PCR* |
| 14 | Patau high risk | | 36 | 159.4 | | 79.2 | 31.2 | 25.4 | Amniocentesis |
| False Negative Case | |  | | |  |  |  |  |  |
| 15 | 47, XN, +21 | | 40 | 170 | | 101 | 34.9 | 12.6 | Amniocentesis |

* Absence of common fetal trisomies was confirmed using stored cord blood which was sampled at a delivery; † No result was obtained with QF-PCR (test failure d/t low DNA amount)

BMI, body mass index; GA, gestational age; QF-PCR, quantitative fluorescent polymerase chain reaction
